# Transmission of *Schistosoma japonicum* in Marshland and Hilly Regions of China: Parasite Population Genetic and Sibship Structure

**DOI:** 10.1371/journal.pntd.0000781

**Published:** 2010-08-03

**Authors:** Da-Bing Lu, James W. Rudge, Tian-Ping Wang, Christl A. Donnelly, Guo-Ren Fang, Joanne P. Webster

**Affiliations:** 1 Department of Infectious Disease Epidemiology, Faculty of Medicine, Imperial College, London, United Kingdom; 2 Anhui Institute of Parasitic Diseases, Wuhu, People's Republic of China; National Institute of Parasitic Diseases China CDC, China

## Abstract

The transmission dynamics of *Schistosoma japonicum* remain poorly understood, as over forty species of mammals are suspected of serving as reservoir hosts. However, knowledge of the population genetic structure and of the full-sibship structuring of parasites at two larval stages will be useful in defining and tracking the transmission pattern between intermediate and definitive hosts. *S. japonicum* larvae were therefore collected in three marshland and three hilly villages in Anhui Province of China across three time points: April and September-October 2006, and April 2007, and then genotyped with six microsatellite markers. Results from the population genetic and sibling relationship analyses of the parasites across two larval stages demonstrated that, within the marshland, parasites from cattle showed higher genetic diversity than from other species; whereas within the hilly region, parasites from dogs and humans displayed higher genetic diversity than those from rodents. Both the extent of gene flow and the estimated proportion of full-sib relationships of parasites between two larval stages indicated that the cercariae identified within intermediate hosts in the marshlands mostly came from cattle, whereas in the hilly areas, they were varied between villages, coming primarily from rodents, dogs or humans. Such results suggest a different transmission process within the hilly region from within the marshlands. Moreover, this is the first time that the sibling relationship analysis was applied to the transmission dynamics for *S. japonicum*.

## Author Summary

*Schistosoma japonicum* involves two obligatory host stages, with asexual reproduction within a molluscan host and sexual reproduction within a mammalian host. Having over 40 species of mammals suspected of being potential reservoirs complicates the transmission patterns. Understanding the complex transmission patterns is further hampered by the ethical and logistical difficulty in sampling adult worms from mammalian hosts. However, the two free-swimming larval stages, cercariae (released from a mollusc and then infective to a mammal) and miracidia (hatched from eggs passed in a mammal's faeces, and then infective to a mollusc), are available, and elucidating the genetic composition of parasites at theses two stages could provide information of infection processes. Here we sampled cercariae during April 2006, miracidia during September-October 2006, and cercariae during April 2007 in three marshland and three hilly villages in Anhui Province of China, and, using microsatellite markers, analyzed the population genetic structure and, for the first time, the familial relationships of parasites at different stages. We found contrasting population structures of parasites, and host species-associated diversities and transmission patterns of parasites between and within two regions. Moreover, we demonstrate that the successful application of sibship analyses to infection process provides an alternative approach to the dissection of transmission dynamics.

## Introduction

A major challenge to the biomedical science in the 21^st^ century is gaining a thorough understanding of the dynamics and evolution of multi-host pathogens [1]. This is especially true for *Schistosoma japonicum*, a multi-host parasite which remains endemic in China [2], the Philippines [3] and parts of Indonesia [4]. In China, after over 50 years of integrated control efforts, including health education, chemotherapy, mollusciciding, environmental management, and sanitation improvement, the disease remains endemic in 110 (out of 433) counties within seven (out of 12) provinces, with an estimated 0.73 million infected humans in 2004 [2]. One feature of the life cycle of this parasite, which distinguishes it from the other human schistosome species, is that a wide spectrum of potential definitive hosts, including over 40 species of domestic and wild mammals across 28 genera, have been identified in China [5]. Based on parasitological survey data, and the availability and abundance of hosts, bovines are generally considered to be the most important reservoirs in terms of chemotherapy-based policy [6]–[8]. Indeed, a recent village-based intervention trial (human and bovine praziquantel treatment *versus* human praziquantel treatment only) confirmed the hypothesis of bovines as the major reservoir host of human schistosomiasis in the lake and marshland regions of China [9]. However, such studies were labour-intensive and costly, and did not take into account any other potential reservoirs, and hence possible transmission pathways, between different definitive host species.

*S. japonicum* involves two obligatory host stages, with asexual reproduction within a molluscan host and sexual reproduction within a mammalian host. Adult worms (males and females) typically reside in the mesenteric venules of the vertebrate host, thus leading to an ethical and logistical difficulty in sampling *S. japonicum* adult worms, particularly from humans and valuable domestic mammals. It is feasible, however, to sample the parasite progeny miracidia, which are hatched from eggs that are passed in a host's faeces. As a single *S. japonicum* miracidium (male or female), after infecting a snail and subsequently undergoing several rounds of asexual reproduction, may give rise to thousands of clonal cercariae, different hosts may be predicted to be infected with the same genotypes of cercariae through water contact. Therefore, under such conditions, one pair of adult worms found in one mammalian host may be genetically identical to a pair found in other hosts. Clonal genotypes of schistosome adult worms, for example *S. mansoni*, have been reported to be present within a single host [10], [11]. On the island of Guadeloupe, the maximum repetition of an identical genotype within a rat was of 22 out of 122 worms genotyped using RAPD markers from a host harbouring 343 schistosomes [10], and among 17 infected rats, about 68.5% of 200 multi-locus genotypes of parasites were shared by two or more rats [12]. As a consequence, the offspring, eggs and subsequent miracidia, from ‘duplicated’ adult worm pairs from the same or different hosts, could be considered as ‘genetic’ siblings even though they belong to different biological parents. Therefore, the approximate worm burdens (the number of genetically unique adult worm pairs) within host individuals and the distribution of given adult worms across host species could be inferred respectively by the estimated number of full-sib families and the full-sibships of the miracidia from all hosts using miracidia genotypes. Moreover, given that most schistosome adult worms within mammals have a life span of between 5 and 30 years [13] and that the eggs from such worms could be produced throughout that period, albeit with a decline in the number of daily discharged eggs over time, the proportion of full-sibships of miracidia (from different host species) and cercariae (from intermediate host snails) could, one may predict, reflect the contribution of each host species to the development of cercariae within the snails.

A storage system for schistosome larval DNA at room temperature with a Whatman FTA® card (Whatman International Ltd., Springfield Mill, James Whatman Way, Maidstone, Kent, UK)- a chemically-treated paper that extracts the DNA from a specimen - has been successfully developed [14], and a single larva DNA, prepared by using a modified HotSHOT method [15], is able to be genotyped with up to 21 microsatellite loci in multi-locus microsatellite analyses [11]. Such methodologies thereby facilitate the sampling of both miracidial and cercarial populations in the field, thus avoiding the introduction of sampling bias through the loss of alleles or genotypes by sampling error or host-induced selection in the subsequent population genetic analyses [14], [16]–[19].

Our previous research [20], [21], based on a two-year epidemiological analyses of *S. japonicum* infection in mammals and snails and/or the gene flow of *S. japonicum* miracidia between definitive host species, have suggested a high possibility of different reservoirs (cattle in marshland areas *versus* rodents in hilly areas) between two regions in Anhui of China. This raises the question of how the parasites were transferred between the suspected reservoir host species and intermediate host snails. As understanding such transmission dynamics could inform the design and focus of control measures, in this study we aimed to elucidate the extent of parasite gene flow between two larval stages (miracidia from mammals and cercariae from snails) based on gene frequencies and the transfer of parasites from definitive to intermediate hosts using, for the first time for this species, sibship analyses. Therefore, we collected *S. japonicum* larvae samples during prevalence surveys implemented in April 2006 (at the beginning of annual transmission season), September–October 2006 (near the end of the annual transmission season) and April 2007 (at the beginning of the next annual transmission season) in three marshland and three hilly villages. We then, by using the previously identified polymorphic microsatellite loci [22], genotyped the three consecutive larval populations and performed population genetic and sibling relationship analyses. The aims were thus to identify any potential difference in transmission dynamics between and within two regions. This study thereby presents, to the authors' knowledge, the first time that full-sibships of parasites at two larval stages were simultaneously sampled and analysed in an effort to improve understanding of the transmission dynamics of macro-parasites.

## Materials and Methods

### Sampling and genotyping of *Schistosoma japonicum* larvae

The details of *S. japonicum* infection in intermediate host snails and definitive host mammals in the sampled villages here have been reported elsewhere [21]. Individual cercariae from infected snails or miracidia hatched from faecal samples from infected definitive hosts were sampled (using a loop for cercariae or a pipette for miracidia) under a binocular microscope. Likewise, the storage of larvae, DNA extraction, and multiplex PCR assay have been reported elsewhere [23]. In brief, ten cercariae per snail for cercariae sampled in each April of 2006 and 2007, and 10–12 miracidia (if available) per individual host for miracidia sampled in September–October of 2006 were genotyped with six primers and then used in molecular analyses. Prior to the population genetic and sibship analyses, the repeated cercariae genotypes within individual snails in either year were reduced to single copies with GENECAP (version 1.2.2) [24], a software designed to find identical genotypes within datasets, as at this stage the parasite undergoes asexual reproduction.

### Population genetic analyses

The population structure of larval parasites for each species of definitive host, or for snails, within each village was defined as containing 2 levels: (1) a parasite infra-population (within-host population), the population of larvae found within an individual host; and (2) a parasite (total) population (within-species population), all larvae found from the individuals of the same species. Wright's *F*-statistics [25] were employed to characterize the structures at the level of infra-populations. *F*_IS_ (*f*) measures the within-host population magnitude of departures from Hardy-Weinberg equilibrium expectations while *F*_ST_ measures the genetic differentiation of parasites between hosts. For each species, the estimator *f* (within-host) and the estimator *F*_ST_ (among hosts within species) were calculated over all loci according to [26] using Genetic Data Analysis software [27]. To assess the significance of the *F*-statistics obtained above, 95% confidence intervals were obtained by bootstrapping over loci with the number of replicates set to 20000. Standard indices of genetic diversity of the parasite (total) population (i.e. a miracidial population for one host species or a cercarial population) were calculated. The observed heterozygosity (Ho) and the expected heterozygosity (He) and their standard deviations were computed with Arlequin [28] by setting permutations at 20000. The numbers of alleles per locus was calculated with HP-RARE1 [29] by taking into account population sizes.

Population pairwise *F*_ST_s, a measure of short-term genetic distance, between pairs of two larval populations, miracidia (from each host species) *vs* cercariae (from snails), were calculated with Arlequin [28] to examine the relationship of the parasites between two larval stages within each village, and their significance was tested by permuting individuals for 20000 times between populations.

### Full-sib analyses

Microsatellite markers, due to their high polymorphism, are particularly well suited to studies of relatedness and kinship assessment [30], [31], and even with few loci, such molecular markers, without *a priori* pedigree information, have been successfully employed to the partitioning of individuals into families [32], [33]. The Colony software (V1.1) [34], which takes into account the typing errors and mutations, has been developed and demonstrated to be able to accurately infer full- and half-sibships from the molecular data of a high error rate. This program implements a maximum likelihood method to assign individuals from a population into full-sib families nested within half-sib families (colonies). It adopts an iterative procedure for updating allele frequencies with reconstructed sibships taken into account, and uses efficient algorithms for calculating the likelihood function and searching for the maximum-likelihood configuration [34]. Colony was applied here, without knowing parental information of *S. japonicum* larvae, to the estimation of full-sib relationships between the sampled larval multi-locus genotypes. All cercariae and miracidia sampled from the same village were pooled and then, by running Colony independently, partitioned simultaneously into full-sib family groups of variable size (group-likelihood approach). As no information on typing errors or mutation rate of the larval samples was available, a small error rate of 1% at each of six loci, as suggested by [34], for both allelic dropout (defined as Class I error in the software) and mutations, false alleles and miscalling (defined as Class II error), was chosen here for the full-sibship analysis of larvae, miracidia and cercariae. Different family configurations were then compared, and the optimal solution of full-sibship reconstruction was chosen when the maximum likelihood estimate was obtained. These were then used to estimate the numbers of genetically unique adult worm pairs within host species or individuals. The transmission of parasites from definitive to intermediate hosts was inferred based on the familial memberships of the parasites between two larval stages, while cross-infection of the parasites, defined as the presence of one genetically unique adult worm pair estimated in more than two host species, was inferred from the distribution of the miracidia of one single configured full-sib family between and among host species.

The mean number of genetically unique adult worm pairs per host and its standard deviation were calculated for each species, and the significant difference among host species was tested with the non-parametric Kruskal-Wallis (for over two independent samples) or the Mann-Whitney U test (for two samples) using SPSS 11.0 software [35].

### Ethics statement

Ethical clearance for the project was obtained from the Scientific Committee at the Anhui Institute of Parasitic Diseases, which is responsible for Schistosomiasis control within the Anhui province where targeted villages are located. As the anti-schistosomiasis work, including health education and testing and treatment of humans, has been conducted in the sampled areas each year, and no detailed personal information is revealed in any subsequent data analyses, oral informed consent, approved by the Scientific Committee and witnessed by local village leaders, was obtained from all adults and from parents or guardians of minors who were involved in the project, and from owners whose domestic animals were sampled, and the names of all participants were then registered by the research team. The approach of sampling stools from domestic animals and from rodents was approved by the Scientific Committee and the work was conducted in accordance with the Anhui Institute of Parasitic Diseases' guidelines for animal husbandry. In brief, domestic animals were released shortly after obtaining stool samples, and those infected with the parasite were given a treatment at 25mg/kg bodyweight if possible. Control advice was also given to the owners of infected dogs or cats. Captured rodents were humanely euthanized with ether in black bags if positive in stool examination; otherwise, they were released at their original point of capture the following day.

## Results

### Population genetic structure of *Schistosoma japonicum* larvae (F-statistics indices)

As seen in Table 1, for *S. japonicum* cercariae in the marshland, a significant within-host population heterozygosity deficiency (*f*), namely a departure from the assumption of Hardy-Weinberg equilibrium, was found in two cases in 2006 with one in Guanghui and the other in Heping. With the exception of cercariae from Longshang and Yuantou in 2007, there was a substantial genetic differentiation among snails observed across the marshland (*F*_ST_ = 0.269–0.471) and the hilly region (*F*_ST_ = 0.103–0.359).

**Table 1:** Standard diversity indices with F-statistics analysis of cercariae samples at levels of villages or individual snails.

	No. of infected snails	No. of cercariae genotyped	No. of alleles per locus	Ho (SD)	He (SD)	*f* (95%CI)	*F*_ST_ (among snails) (95%CI)
Guanghui (M)							
2006	34	85	8.17	0.333(0.145)	0.675(0.201)	0.335(0.051,0.505)	0.269(0.203,0.319)
2007	39	140	9.05	0.400(0.148)	0.675(0.198)	0.089(−0.164,0.304)	0.413(0.227,0.559)
Heping (M)							
2006	39	79	7.54	0.280(0.141)	0.663(0.216)	0.348(0.098,0.576)	0.360(0.292,0.421)
Xingzhuang (M)							
2006	31	81	8.61	0.330(0.179)	0.700(0.225)	0.314(−0.041,0.604)	0.323(0.258,0.372)
2007	27	72	9.14	0.384(0.150)	0.714(0.201)	0.103(−0.110,0.334)	0.471(0.251,0.639)
Longquan (H)							
2006	27	45	6.05	0.313(0.219)	0.584(0.250)	0.264(−0.014,0.510)	0.284(0.220,0.372)
2007	30	67	5.93	0.391(0.214)	0.603(0.246)	0.023(−0.269,0.289)	0.359(0.120,0.582)
Longshang (H)							
2006	27	46	5.30	0.297(0.259)	0.552(0.244)	0.240(−0.183,0.703)	0.303(0.133,0.396)
2007	27	53	3.55	0.435(0.329)	0.481(0.300)	−0.298(−0.454,0.006)	0.103(−0.057,0.373)
Yuantou (H)							
2006	23	31	4.02	0.357(0.293)	0.434(0.304)	0.030(−0.376,0.440)	0.159(0.023,0.246)
2007	34	85	4.34	0.396(0.325)	0.467(0.321)	−0.199(−0.536,0.152)	0.158(−0.210,0.435)

Note: No. of alleles per locus, with rarefaction including 38 genes per sample; Ho, observed heterozygosity over 6 loci; He, expected heterozygosity over 6 loci; M, marshland; and H, hilly region.

As displayed in Table 2, among the three marshland villages, the genetic structures of the miracidia from all species (excluding humans) studied here were all very similar, with high gene flow of parasites among hosts within species (*F*_ST_≤0.021) and of significant within-host population heterozygote deficiency (*f*). However, within the hilly region, a marked within-host heterozygote deficiency was only found in parasites from dogs in Longquan and from humans in Yuantou. For all host species, with the exception of dogs in Longquan, the *F*_ST_ values ranged from 0.094 to 0.274, indicating low gene flow of parasites among hosts. It was also noted that most of the 95% confidence intervals of the *F*_ST_ values were much wider in the hilly than in the marshy villages.

**Table 2:** Standard diversity indices with F-statistics analysis of miracidia samples at levels of species or individual hosts.

	No of hosts	No. of miracidia genotyped	No. of alleles per locus	Ho (SD)	He (SD)	*f* (95%CI)	*F*_ST_ (among hosts) (95%CI)
Guanghui (M)							
Buffalo	2	22	4.54	0.294(0.172)	0.630(0.204)	0.542(0.324,0.739)	−0.007(−0.040,0.028)
Cattle	15	168	4.71	0.387(0.116)	0.643(0.201)	0.387(0.167,0.533)	0.021(0.004,0.034)
Dogs	2	21	4.80	0.402(0.119)	0.642(0.234)	0.383(0.196,0.524)	−0.007(−0.047,0.043)
Heping (M)							
Cattle	3	30	4.77	0.414(0.172)	0.652(0.221)	0.363(0.133,0.539)	0.016(−0.023,0.065)
Xingzhuang (M)							
Cattle	13	150	4.93	0.395(0.114)	0.664(0.204)	0.396(0.227,0.525)	0.016(0.005,0.031)
Goats	8	83	4.64	0.396(0.085)	0.653(0.202)	0.397(0.253,0.491)	−0.004(−0.018,0.012)
Humans	1	9	2.59	0.519(0.175)	0.517(0.193)	−0.004(−0.247,0.182)	-
Longquan (H)							
Cats	1	7	1.83	0.333(0.375)	0.294(0.312)	−0.148(−0.333, 0.217)	-
Dogs	8	90	3.85	0.379(0.232)	0.561(0.276)	0.306(0.017, 0.546)	0.034(0.010, 0.057)
Rodents	6	67	3.53	0.429(0.213)	0.558(0.261)	0.094(−0.135,0.510)	0.180(0.121,0.251)
Longshang (H)							
Dogs	1	1	1.5	0.5	0.5	−1.0	-
Humans	5	47	4.42	0.417(0.069)	0.666(0.191)	0.187(−0.026,0.337)	0.274(0.204,0.345)
Rodents	5	55	3.03	0.373(0.333)	0.445(0.331)	0.067(−0.133,0.366)	0.124(0.068,0.165)
Yuantou (H)							
Dogs	4	31	3.13	0.384(0.231)	0.500(0.286)	0.148(−0.146,0.373)	0.138(0.039,0.242)
Humans	3	36	3.71	0.426(0.219)	0.551(0.263)	0.175(0.074,0.288)	0.094(0.005,0.164)
Rodents	2	23	2.4	0.447(0.356)	0.425(0.301	−0.226(−0.546,0.014)	0.238(0.059,0.368)

Note: No. of alleles per locus, with rarefaction including 12 genes per sample; Ho, observed heterozygosity over 6 loci; He, expected heterozygosity over 6 loci; M, marshland; and H, hilly region.

### Allele-based genetic diversity

As displayed in Table 1, both the allelic diversity (the number of alleles per locus) and expected heterozygosity (He) of cercarial populations were higher in the marshland than in the hilly region. For example, the total number of alleles per locus ranged from 7.5 to 9.1 among the marshland villages, whereas it was between 3.6 and 6.1 among the hilly ones.

From Table 2, within the marshland, the miracidial populations from cattle or dogs in Guanghui and from cattle in Xingzhuang exhibited higher genetic diversity than the populations from the other species. However, in the hilly villages the highest allele richness and expected heterozygosity were always found in miracidial populations from humans or dogs rather than from rodents.

### Genotype-based genetic diversity

In the marshland, the highest infection prevalence in snails was 8.1% in Guanghui in 2007, while the highest value of 1.4% in the hilly region was found in Yuantou. The mean abundance of *S. japonicum* infection within snails (the number of cercariae genotypes per infected snail) was higher in the marshland than in the hilly region in both years (see Table 3). In both marshland and hilly villages, the observed distribution of cercariae genotypes differed significantly from expected abundance values under the assumption of random distribution (Poisson goodness-of-fit tests, all p<0.001). Within the hilly region, in Longquan and Yuantou in 2006, the observed distribution of cercariae genotypes showed an over-dispersion (negative binomial distribution goodness-of-fit tests, p = 0.46 in Longquan, and p = 0.05 in Yuantou).

**Table 3:** Distribution of *S. japonicum* infections in snails in 2006 and 2007.

Villages	Years	Infected snails (%)	No. of total cercariae genotyped	Mean abundance*	No. parasite genotypes per snail (genotyping 10 cercariae per snail)									p†	p‡
					1	2	3	4	5	6	7	8	9		
Guanghui (M)	2006	34(5.21)	85	2.50	15	4	8	4	0	1	1	0	1	<0.001	0.020
	2007	39(8.11)	140	3.59	4	10	9	5	3	4	2	2	0	<0.001	<0.001
Heping (M)	2006	39(2.77)	79	2.03	15	13	7	3	1	0	0	0	0	<0.001^+^	0.003
	2007	0(0/1243)													
Xingzhuang (M)	2006	31(2.70)	81	2.61	7	10	6	4	4	0	0	0	0	<0.001^+^	<0.001
	2007	27(2.55)	72	2.67	4	12	3	6	1	1	0	0	0	<0.001^+^	<0.001
Longquan (H)	2006	27(0.74)	45	1.67	18	6	0	1	1	1	0	0	0	<0.001^+^	0.456
	2007	30(1.32)	67	2.23	9	11	5	4	1	0	0	0	0	<0.001^+^	<0.001
Longshang (H)	2006	27(1.41)	46	1.70	14	9	2	2	0	0	0	0	0	<0.001^+^	0.039
	2007	27(1.13)	53	1.96	7	14	6	0	0	0	0	0	0	<0.001^+^	<0.001
Yuantou (H)	2006	23(1.44)	31	1.35	16	6	1	0	0	0	0	0	0	<0.001^+^	0.051^+^
	2007	34(1.44)	85	2.50	6	14	7	5	2	0	0	0	0	<0.001^+^	<0.001

Note: * mean abundance is the number of cercariae genotypes per infected snail; † p is the p-value of Poisson distribution goodness-of-fit test; ‡ p is the p-value of Negative binomial distribution goodness-of-fit test; +, for one cell with an expected value less than 3 in calculation of the χ^2^ value under a given distribution. M, for marshland; and H, for hilly region.

The number of genetically unique adult worm pairs within a host individual was inferred from the reconstructed families of the miracidia within that host, from which a mean value for each host species was further calculated. As shown in Table 4, within the marshland, more genetically unique adult worm pairs per host were inferred in cattle than in any other species in each village, with the exception of Heping where only infected cattle were found. In Longquan within the hilly region, dogs harboured a mean number of eight pairs of worms per host, higher than that found within rodents or cats. In the case of Yuantou, a dog, plus a human, appeared to be infected with more unique genotypes of parasites than a rodent. In Longshang a rodent seemed to be infected with more parasites than a human did. However, a non-significant difference in such index was seen in both marshland and hilly villages. Generally, definitive host individuals seemed to harbour more worm pairs in the marshland than in the hilly region (see Table 4).

**Table 4:** Estimated genetically unique adult worm pairs within individual definitive hosts for each host species.

Village	Host species	No. host individuals	Mean no. analyzed miracidia (SD)	Mean no. estimated adult worm pairs (SD)
Guanghui (M)	Buffalo	2	11 (0)	9.5 (0.71)
	Cattle	15	11 (1)	11 (1.50)
	Dogs	2	10.5 (0.71)	10 (1.41)
Kruskal-Wallis Test, χ^2^ = 0.30, df = 2, p = 0.86				
Heping (M)	Cattle	3	9.3(1.15)	8 (1.73)
Xingzhuang (M)	Cattle	13	11.5(0.89)	10.2(1.46)
	Goats	8	10.4(3.42)	8.3(2.92)
	Humans	1	9	2
Kruskal-Wallis Test, χ^2^ = 5.20, d.f. = 2, p = 0.07				
Longquan (H)	Cats	1	7	1
	Dogs	8	11.1(0.64)	8(1.51)
	Rodents	6	10.5(1.05)	5.33(2.80)
Kruskal-Wallis Test, χ^2^ = 5.42, d.f. = 2, p = 0.07				
Longshang (H)	Humans	5	9.2(1.6)	3.2(1.47)
	Dogs	1	1	1
	Rodents	5	10.2(0.75)	5(2.10)
Kruskal-Wallis Test, χ^2^ = 3.77, d.f. = 2, p = 0.15				
Yuantou (H)	Humans	3	12(1)	6(0)
	Dogs	4	7.8(3.50)	5.5(3.11)
	Rodents	2	11(0)	3(2.83)
Kruskal-Wallis Test, χ^2^ = 2.28, d.f. = 2, p = 0.32				
Difference between M and H, Mann-Whitney U test, p<0.001, 2 tailed				

Note: M, for marshland, and H, for hilly region.

As shown in Table 5, out of a total of 82 genetically unique worm pairs estimated from miracidial samples from cattle, water buffalo and dogs in Guanghui, 76 (92.7%) were found in cattle and 49 of these were found exclusively in cattle. In Xingzhuang, a total of 88 genetically unique adult worm pairs were estimated, and the number of the total pairs in cattle was 75(85.2%), with 21(23.9%) found both in cattle and goats, and one pair (1.1%) found both in cattle and humans. In Heping, 20 unique pairs of adult worms were identified in cattle, the only host species found in the village.

**Table 5:** Distribution of genetically unique adult worm pairs (%) within and across host species.

Village	No. of adult worm pairs	No. of adult worm pairs exclusive to each host species			No. of adult worm pairs <1?show=[to]?>shared among host species			
		Buffalo	Cattle	Dogs	Buffalo and cattle	Buffalo and dogs	Cattle and dogs	Buffalo, cattle and dogs
Guanghui (M)	82	2(2.4)	49(59.8)	3(3.7)	11(13.4)	1(1.2)	14(17.1)	2(2.4)
			Cattle					
Heping (M)	20		20					
		Humans	Cattle	Goats	Humans and cattle	Cattle and goats		
Xingzhuang(M)	88	1(1.1)	53(60.2)	12(13.6)	1(1.1)	21(23.9)		
		Cats	Rodents	Dogs	Dogs and rodents			
Longquan (H)	41	1(2.4)	4(9.8)	15(36.6)	21(51.2)			
		Humans	Rodents		Humans and dogs	Humans and rodents		
Longshang (H)	24	9(37.5)	10(41.7)		1(4.2)	4(16.7)		
		Humans	Rodents	Dogs	Humans and dogs	Dogs and rodents	Humans, dogs and rodents	
Yuantou (H)	19	4(21.1)	1(5.3)	1(5.3)	8(42.1)	3(15.8)	2(10.5)	

Note: M, marshland; and H, hilly region.

In the hilly village Longquan, 41 genetically unique worm pairs were inferred from miracidia samples obtained from dogs, cats and rodents, of which 36 (87.8%) and 25 (61.0%) were inferred in dogs and rodents, respectively. In Longshang, only 24 pairs were estimated, with 14 pairs harboured in either of humans and rodents. In Yuantou, 19 pairs were estimated, out of which the numbers of pairs in humans, dogs, and rodents were 14, 12 and 6, respectively (see Table 5).

### Allele-based transmission of parasites between two larval stages

Table 6 illustrates that, across three marshland villages, the smallest estimates of *F*_ST_ (or highest gene flow) were observed between the miracidial populations from cattle and the cercarial populations from snails, ranging from 0.002 to 0.017 across both years. In the hilly region, as illustrated in Table 6, in Longquan, the highest gene flow was seen between the cercarial population and the miracidial populations from dogs or rodents. In Longshang, such close relatedness was seen only between the cercarial population and the miracidia from rodents. In Yuantou, the smallest estimate was found between the cercarial population and the miracidia from dogs, with the second between the cercariae and those from humans.

**Table 6:** Pairwise F_ST_s between cercarial populations from snails and miracidial populations from each host species.

	Humans	Goats	Buffalo	Cattle	Cats	Dogs	Rodents
Guanghui (M)							
Snails(2006)			0.010	0.017**		0.041**	
Snails(2007)			0.014	0.008*		0.016	
Heping (M)							
Snails(2006)				0.011			
Xingzhuang (M)							
Snails(2006)	0.129**	0.023**		0.002			
Snails(2007)	0.136**	0.012**		0.008*			
Longquan (H)							
Snails(2006)					0.459**	−0.010	−0.034
Snails(2007)					0.428**	0.003	0.018**
Longshang (H)							
Snails(2006)	0.114**					0.294*	0.043**
Snails(2007)	0.154**					0.381*	−0.006
Yuantou (H)							
Snails(2006)	0.054**					0.043**	0.133**
Snails(2007)	0.056**					0.032**	0.115**

Note: * p<0.05; ** p<0.01. M, marshland; and H, hilly region.

### Genotype-based transmission from definitive to intermediate hosts

The transmission of parasites from definitive to intermediate hosts was inferred based on the familial memberships of miracidia from mammals and of cercariae from molluscs. As displayed in Table 7, of the 85 and 140 cercariae genotypes identified from the infected snails in Guanghui in 2006 and 2007, respectively, 56 (65.9%) and 89 (63.6%) were estimated to be from cattle. This was also true in the case of Xingzhuang, with a majority of cercariae (71.6% in 2006 and 59.7% in 2007) inferred to have originated from cattle. However, it was also noted that it was not possible to trace the resource reservoirs of 27.1–31.4% of the cercariae in Guanghui and of 13.9–27.2% of the cercariae in Xingzhuang, as no miracidia were inferred to be sibs to any of those cercariae. In Heping, only 36 out of 79 cercariae sampled in 2006 were inferred to have originated from cattle. This could be potentially attributed to the inaccessibility for the cattle to one of two infectious habitats where infected snails were sampled.

**Table 7:** The number and proportion (%) of cercariae estimated from definitive hosts.

Village	Year	No. of cercariae	From single host species only			From potential multiple host species			Unknown
			Buffalo	Cattle	Dogs	Buffalo/cattle	Buffalo/dogs	Cattle/dogs	
Guanghui (M)	2006	85	2(2.4)	44(51.8)	2(2.4)	7(8.2)	2(2.4)	5(5.9)	23(27.1)
	2007	140	3(2.1)	67(47.9)	4(2.9)	10(7.1)	0	12(8.6)	44(31.4)
				Cattle					
Heping (M)	2006	79		36(45.6)					43(54.4)
			Humans	Cattle	Goats	Humans/cattle	Cattle/goats		
Xingzhuang (M)	2006	81	0	46(56.8)	1(1.2)	0	12(14.8)		22(27.2)
	2007	72	2(2.8)	32(44.4)	17(23.6)	1(1.4)	10(13.9)		10(13.9)
			Cats	Rodents	Dogs	Dogs/Rodents			
Longquan (H)	2006	45	0	3(6.7)	10(22.2)	28(62.2)			4(8.9)
	2007	67	0	2(3.0)	26(38.8)	30(44.8)			9(13.4)
			Humans	Rodents		Humans/dogs	Humans/rodents		
Longshang (H)	2006	46	6(13.0)	25(54.3)		0	2(4.3)		13(28.3)
	2007	53	1(1.9)	37(69.8)		0	8(15.1)		7(13.2)
			Humans	Rodents	Dogs	Humans/dogs	Dogs/rodents	Humans/dogs/rodents	
Yuantou (H)	2006	31	2(6.5)	0	4(12.9)	5(16.1)	4(12.9)	1(3.2)	15(48.4)
	2007	85	11(12.9)	4(4.7)	15(17.6)	8(9.4)	1(1.2)	2(2.4)	44(51.8)

Note: M, for marshland; and H, for hilly region.

In the hilly village Longquan, there was evidence to suggest that parasites may be transmitted primarily from dogs and/or rodents to intermediate host snails, as most of the cercariae were successfully inferred from the two species. In Longshang, a substantial proportion of cercariae, up to 58.6% in 2006 and 84.9% in 2007, were estimated to come from rodents. However, in Yuantou a considerable number of cercariae (48.4–51.8%) were not traced back to their original reservoirs, with the remainder attributed to dogs, humans and/or rodents (see Table 7).

## Discussion

The results from the population genetic and sibling relationship analyses of parasites at two larval stages demonstrated contrasting population genetic structures of parasites between the two regions, and the definitive host-associated genetic diversities and transmission patterns of parasites between and within two regions. The observations from both the gene flow and the estimated proportion of full-sib relationships of parasites between two larval stages indicated that the cercariae identified within snails in the marshland may mostly come from cattle, whereas in the hilly areas, they were varied between villages, coming from rodents, dogs or humans.

An adult within-host population, i.e. the group of the parasites present within one host at a particular time [36], and then a derived miracidia within-host population, constitute the primary levels of population parasite fragmentation [37], [38]. In this study, within marshland villages the F-statistics analyses, measuring the within-(*f*) and among-host (*F*_ST_) genetic variance of miracidial populations each from species of cattle, water buffalo, goats or dogs, showed a similar pattern, consistent with high gene flow among hosts and high within-host inbreeding. As water contact activities for mammals may vary from species to species, or even from individual to individual, the observed high gene flow among hosts and the high inbreeding within hosts would suggest that, within each village all host individuals may each sample a various number of cercariae originating from the same parasite population, namely the common infection ‘foci’. As previously reported [21], in the marshland snails are more widely dispersed across considerably large marshy areas of land, ranging from 10, 000 to 60, 000 m^2^ among villages. An infection focus was found both in Guanghui and Xingzhuang, to which humans and domestic animals have free accessibility. In Heping, there were two infection foci, separated by a Yangtze River branch. It would be suspected that these foci each may comprise a large ‘well-mixed’ parasite population, in terms of allelic or genotypic composition. Within the hilly region, in contrast, most miracidial populations from rodents (with lower mobility), dogs or humans (with higher mobility) showed a pattern of low gene flow among hosts and of no or low within-host inbreeding. This may mainly due to the existence of several separated ‘less-mixed’ foci within each of the three villages. As previously reported [21], 5, 3 and 5 infection habitats were respectively found in Longquan, Longshang and Yuantou in 2006, and 5, 10 and 2 infection habitats respectively in 2007. Another explanation could partially arise from the biological traits of the different parasite strains between two habitats [23], which could have lead to the differential infection profile in mammals between two regions.

The highest genetic diversity (genotype-based) was observed in miracidial populations from cattle than from any other mammals in each marshland village; whereas within hilly villages it was observed in parasites from dogs or humans, instead of rodents. Such difference among species within each village could be, to some extent, explained by several factors. First, definitive hosts from the more dispersing species or the species with a larger home range, for example, humans, dogs, and bovines compared to rodents, could harvest a higher number of genetically distinct larval parasites. As predicted, the results of this study did show that hosts with high movement, such as cattle in the marshland and dogs or humans in the hilly region, appeared to harbour the highest numbers of genetically unique adult worm pairs. Second, species with longer life-spans are likely to obtain more genetically diverse infections then species with shorter life-spans. In the marshland the highest estimated number of worm pairs were observed in cattle with an average of more than 10 years of lifespan, whereas in the hilly region the lowest in rodents with an average of less than 2 years of lifespan. Although humans may have been exposed over an even longer time period than the cattle, a lower index in humans at the time point of the survey could have resulted from the annual testing and treatment required of people in such endemic areas [39]. Finally, host immunity could have a great influence on the population growth within hosts [40]. From this study, in Guanghui (marshland), non-significantly higher numbers of genetically unique adult worm pairs were estimated in cattle than in water buffalo, partly due to the differential immunity against *S. japonicum* between both [41].

The higher genetic diversity (the number of alleles per locus and expected heterozygosity) of cercarial populations at the level of villages in the marshland than in the hilly region was consistent with those observed for miracidial populations (parasites from cattle in the marshland *vs* parasites from rodents in the hilly region). Multiple infection of schistosomes in snails has been well documented for *S. mansoni* and *S. haematobium* in various ecological and epidemiological transmission settings [42]–[46], from which the number of cercarial genotypes per snail, on average, ranged from 1.1 to 6.2. In this study, it was more common for snails to harbour multiple parasite genotypes in the marshland than in the hilly region. Over-dispersion of *S. japonicum* infections among snails was found in two hilly villages. The difference in the proportion of multiple infections and the extent of over-dispersion of infections among snails between the two regions could, on one hand, result from heterogeneities in the exposure, mainly associated with definitive host excreting movements. In the marshland, for example, bovines with a longer life span seem to be ideal ‘genetic mixing bowls’ for the parasites and then may cause the large ‘well-mixed’ infection foci via daily herding and discharging huge amounts of faeces in snail infested areas; whereas in the hilly region, it would be suspected that such separated ‘less mixed’ foci could be attributed to small territory-bound and short-lived rodents. On the other hand, multiple infection within snails could also be an artifact caused by the somatic mutation for *S. japonicum* at the asexual stage [47], as we indeed observed quite a few of cercarial genotypes within single snails differed in one locus only (data not shown). This was partially supported by the estimation of the parasites from mammals to snails, in which a number of cercariae within each village could not be traced back to their original definitive hosts. Population genetic structures of cercariae thus differed between or even within regions mainly due to a possible difference in the proportion of infections within a snail caused by sibling miracidia, non-related miracidia, somatic mutation of cercariae, or indeed a combination of all of the above.

*Schistosoma japonicum* cercariae from a given infection focus within each sampled village may not vary, in terms of genotypes or genotype composition, rapidly within a short period of a few days, weeks or months due to the relative longevity of the same snails consistent over these time periods [48], [49]. Cross–infections among host species may therefore occur but their frequencies mainly depend on their accessibility of the focus. In this study, in the marshland, all the observed mammals have free access to grasslands and shallow water bodies along the banks, with bovines usually grazing in the morning and dogs roaming randomly there at will. In the hilly areas, infected snail habitats are distributed along ditches around patched rice fields, which each belong to a different farmer. It is thus very likely that, in the hilly region, the owners, their dogs and nearby rodents, may contact the same pool of cercariae. From the epidemiological point of view, within each village the number of genetically unique worm pairs shared between or among mammal species, plus the number of worm pairs estimated within host species each, could have implications in terms of potential main reservoirs in the transmission. Indeed, in marshland villages, cattle seem to be main contributors, whereas within the hilly region the main contributors may vary with villages. It is worth mentioning that for cattle in the marshland the mean number of analyzed miracidia per host is almost equal to the mean number of estimated adult worm pairs per host, suggesting that many more larvae remain to be sampled for estimation of adult worms. However, in Guanghui for example, a total of only 82 genetically (rather than 165 (15×11 for cattle) or 204 (15×11 for cattle, 2×9.5 for buffalo and 2×10 for dogs)) unique adult worm pairs were inferred from the offspring of 211 miracidia from all host species, indicating that the estimated adult worms could be, at least, one part of the main circulation parasites, as 27 (out of 82) pairs were observed to be common between/among host species.

The contrasting genetic structures of miracidial populations between two regions strongly indicated different main reservoirs between them. Within the marshland region, a higher genetic diversity of schistosomes in cattle than in other species also indicated a possibility of cattle as a main reservoir in such areas. This is further supported by both the observed Pairwise *F*_ST_s and the sibling relationship analyses. However, in the hilly region, based on genetic diversities, Pairwise *F*_ST_s, and full-sib relationships of parasites, the main reservoirs may be dogs and rodents in Longquan, rodents in Longshang, and dogs and humans (although not completely supported by sibling analyses) in Yuantou. Annual selective chemotherapy, health education and improvement in sanitation may have been greatly reduced the role of humans in the transmission [50]. Recent evidence suggests a role of dogs in the transmission of the *S. japonicum* in the Philippines [51], [52]. Therefore, the transmission pattern of *S. japonicum* within such hilly areas could be reasonably inferred as follows: ‘clonal’ rodents maintain the parasite life cycle within their own territory, which may include one or more infection foci; with the existence of such several territories in each village, dogs get infected in one or more and, due to higher mobility, spread the parasites among territories; dogs could also get infected outside the village and then spread them; new infection territories would develop when immigrant parasites infect and establish within local rodents.

There are, however, two potential limitations inherent within this study. One is with regard to sample size [53] and associated effective population size [54] when comparing genetic diversities of parasites. Indeed, the analyzed larval samples varied with villages and even (for miracidia) varied with host species. However, such sample sizes mainly depend on the number of infected hosts available. Since the accuracy of relatedness estimates from sibling analyses is affected by the number of loci, the number of alleles per locus [32], and true full-sib family sizes [55], considering that only six markers, indeed a moderate number of markers, were analysed in this study for inferring familial relationship of parasites, it would be recommended that more highly polymorphic microsatellite markers should be developed and employed in such analyses.

Nevertheless, this is the first time that the potential number of genetically unique adult worm pairs within mammal hosts has been estimated using their offspring miracidia, although the concept of estimating such a parameter has been raised by Criscione and colleagues [56], and that family memberships of parasites between/within two larval stages has been employed to elucidate the transmission pattern. The observations from both the gene flow and the estimated proportion of full-sib relationships of parasites between two larval stages indicated that in the marshland cercariae identified within snails most likely come from cattle, whereas in the hilly areas, they most likely come from rodents, dogs or humans. Dogs, with higher mobility than rodents, might play a role in spreading parasites among territories, leading to the epidemic or outbreak of the diseases. These results have practical implications in terms of targeted control, particularly in the hilly region in which control strategy should focus on dogs as well as snails.
